# The association between arterial stiffness and left ventricular filling pressure in an apparently healthy Korean population

**DOI:** 10.1186/1476-7120-11-2

**Published:** 2013-01-09

**Authors:** Hack-Lyoung Kim, Moon-Sun Im, Jae-Bin Seo, Woo-Young Chung, Sang-Hyun Kim, Myung-A Kim, Joo-Hee Zo

**Affiliations:** 1 Cardiovascular Center, Seoul National University Boramae Medical Center, Seoul, Korea; 2 Department of Internal Medicine, Seoul National University College of Medicine, Seoul National University Boramae Medical Center, 39 Boramae-gil, Seoul, Dongjak-gu, 156-707, Korea

**Keywords:** Arterial stiffness, Association, Left ventricular filling pressure, Pulse wave velocity

## Abstract

**Background:**

The aim of this study is to investigate the association between arterial stiffness and left ventricular filling pressure in an apparently healthy Korean population.

**Methods:**

A total of 115 healthy subjects without known cardiovascular risk factors or overt heart disease who underwent both transthoracic echocardiography and brachial-ankle pulse wave velocity (baPWV) measurement at the same day during their routine check-ups were analyzed.

**Results:**

The mean age of study subjects was 52.8 ± 8.4 years, and 78 (67.8%) were men. The mean baPWV value was 1,325 ± 185 cm/s. Study subjects were divided into 3 groups according to E/E’ value: subjects with E/E’ < 8, 8–12.9 and E/E’ ≥ 13. As E/E’ increased, baPWV value increased gradually: baPWV in subjects with E/E’ < 8, E/E’ 8–12.9 and E/E’ ≥ 13, were 1,261 ± 163, 1,345 ± 169, 1,569 ± 232 cm/s, respectively (*p* < 0.001). In multiple linear regression analyses, baPWV was significantly associated with E/E’ (*β* = 0.371*, p* < 0.001*)* after controlling confounders including age, sex and body mass index. In receiver-operating characteristic (ROC) curve analysis, the sensitivity and specificity for detection of E/E’ ≥ 10 were 78.6% and 59.8%, respectively with mean baPWV of 1,282 cm/s as the cut off value. The discriminatory capacity for predicting E/E’ ≥ 10 was improved from an area under the ROC curve of 0.646 with age alone to 0.734 when baPWV was added (*p* < 0.001).

**Conclusions:**

There is a significant association between baPWV and E/E’ in an apparently healthy Korean population. BaPWV is useful as a simple and non-invasive method for early detection of increased LV filling pressure among these people.

## Background

Stiffened artery increases pulse pressure leading to left ventricular hypertrophy and coronary artery disease [1]. Arterial stiffness has been reported to be associated with cardiovascular morbidity and mortality [2,3].

There are several methods to estimate arterial stiffness. Among them, pulse wave velocity (PWV) is generally accepted as the most simple, non-invasive and validated indicator of arterial stiffness [4,5]. Recently, the brachial-ankle PWV (baPWV) measurement, which is easy to perform, has become available in clinical practice [6]. Its validity and usefulness were proven in various clinical settings [7-12] and meta-analysis [3,13].

There have been several studies on the association between arterial stiffness and left ventricular (LV) diastolic performance, suggesting that arterial stiffness may potentially contribute to the development of LV diastolic dysfunction through increased pulse pressure and LV afterload [14-20]. However, populations with special medical conditions such as hypertension [14,15,18-20], diabetes [14,17], or renal failure [16] were investigated in those studies. Therefore, the relation of arterial stiffness to diastolic performance in a healthy population remains unclear. In addition, E/E’, the most important and reliable parameter of LV diastolic function, has not been focused in those studies. Therefore, this study was conducted to investigate the association between baPWV and E/E’ in a healthy population.

## Methods

### Study subjects and protocols

Between January 2010 and May 2012, a total of 219 consecutive subjects who underwent both transthoracic echocardiography and baPWV measurement at the same day during their routine check-ups were retrospectively reviewed. All study subjects were recruited in one cardiovascular center (Boramae Medical Center, Seoul, Korea). One hundred and four subjects who had one or more known cardiovascular risk factors including hypertension, diabetes and dyslipidemia (n = 68), coronary artery disease (n = 2), stroke (n = 1), LV ejection fraction of < 50% in echocardiography (n = 1), renal disease with estimated glomerular filtration rate < 60 ml/min/1.73 m^2^ (n = 6), atrial fibrillation (n = 3), and unavailable information on E/E’ (n = 23) were excluded. Finally, 115 apparently healthy subjects without known cardiovascular risk factors or overt cardiovascular disease were analyzed in this study. Approval for the study protocol was obtained from the Institutional Review Board of Boramae Medical Center (Seoul, Korea). Informed consent was waved due to routine nature of the information collected and retrospective study design.

### BaPWV

Patients were examined in the supine position after for 5 or more minutes rest. BaPWV were measured using a volume-plethysmographic apparatus (VP-1000; Colin Co. Ltd., Komaki, Japan) in accordance with the manufacturer’s recommendations. Cuffs were wrapped on both brachials and ankles. Phonogram, pulse volume waveform, blood pressure and heart rate were recorded simultaneously. PWV was calculated by measuring the time for the pulse wave to travel between the brachial and posterior tibial arteries. The mean value between left and right baPWV was used for study analysis. All measurements were performed by the same experienced operator blinded to patients’ information.

### Echocardiography

Transthoracic echocardiography was performed according to recommendations of current guidelines [21] using commercially available equipments (Sequoia, Siemens Medical Solutions or Vivid 7, GE Medical Systems). M-mode echocardiographic features were used for measurement of LV and left atrial dimension, with an LV ejection fraction from parasternal short axis view. The peak early transmitral filling velocity during early diastole (E), late diastole (A) and deceleration time (DT) were imaged in the apical four chamber view at the tip of the mitral leaflets. Color-coded tissue Doppler imaging (TDI) was applied to the apical four chamber view to determine mean early (E’) and late (A’) velocity at the septal mitral annulus. Two experienced cardiosonographers performed echocardiography. Interobserver agreement of E/E’ was evaluated by Spearman’s correlation among 50 subjects. The correlation coefficient was 0.893 in our laboratory.

### Statistical analysis

Continuous variables were presented as mean ± standard deviation (SD), and categorical variables were expressed as percentages. Continuous variables were compared using Student *t* test and categorical variables were compared using chi-square test. Subjects were grouped according to E/E’ values (group with E/E’ < 8, 8–12.9 and ≥ 13) [22] during univariate comparisons. Multiple linear regression analysis was performed to assess independent association between baPWV and E/E’ by adjustment of age, sex and body mass index. Gender stratified analysis was also performed in this multivariable model. Pearson’s correlation method was used for assessing the relationship between baPWV and E/E’. To assess cut off value of baPWV as a predictor of E/E’ ≥ 10, receiver operating characteristic (ROC) curve analysis was used. Probability data from age and baPWV was generated using logistic regression analysis, and used it to assess whether baPWV had an incremental value over age for predicting E/E’ ≥ 10 in ROC curve analysis [23]. A *p* value of < 0.05 was considered statistically significant. All statistical analyses were conducted using SPSS 18.0 (Chicago, IL, USA).

## Results

Baseline characteristics of study subjects are summarized in Table 1. The mean age was 52.8 ± 8.4 years, and 78 (67.8%) were men. The mean baPWV value was 1,325 ± 185 cm/s. Study subjects were divided into 3 groups according to E/E’ value: subjects with E/E’ < 8, 8–12.9 and E/E’ ≥ 13 [22]. Subjects with higher E/E’ were older (*p* = 0.009) and female predominant (*p* = 0.026). Systolic and diastolic blood pressure were higher in subjects with higher E/E’ than subjects with lower E/E’ (*p* < 0.05 for each). As E/E’ increased, baPWV value increased gradually: baPWV in subjects with E/E’ < 8, E/E’ 8–12.9 and E/E’ ≥ 13, were 1,261 ± 163, 1,345 ± 169, 1,569 ± 232 cm/s, respectively (*p* < 0.001).

**Table 1:** Baseline clinical characteristics of study subjects according to E/E’

**Characteristic**	**Total (n = 115)**	**E/E’ < 8 (n = 51)**	**E/E’, 8–12.9 (n = 56)**	**E/E’ ≥ 13 (n = 8)**	*p *value^*^
Age, years	52.8 ± 8.4	50.6 ± 9.2	53.9 ± 6.7	59.7 ± 11.5	0.009
Male sex, n (%)	78 (67.8)	38 (74.5)	38 (67.9)	2 (25.0)	0.026
Body mass index, kg/m^2^	24.1 ± 2.9	23.1 ± 3.0	24.7 ± 2.6	26.4 ± 3.3	0.002
Systolic blood pressure, mmHg	120 ± 14	115 ± 15	123 ± 12	130 ± 15	0.006
Diastolic blood pressure, mmHg	73.5 ± 10.6	70.5 ± 11.7	76.0 ± 8.8	75.3 ± 10.4	0.022
Total cholesterol, mg/dL	199 ± 31	193 ± 32	204 ± 28	203 ± 39	0.228
Triglyceride, mg/dL	106 ± 57	98 ± 51	113 ± 63	84 ± 36	0.232
LDL-choleterol, mg/dL	128 ± 29	125 ± 28	131 ± 28	134 ± 35	0.449
HDL cholesterol, mg/dL	50.2 ± 11.4	50.8 ± 9.9	49.6 ± 12.8	52.0 ± 10.5	0.786
Fasting plasma glucose, mg/dL	92.3 ± 12.3	90.1 ± 10.6	94.9 ± 13.4	85.8 ± 9.1	0.040
Left ventricular ejection fraction, %	66.6 ± 5.9	66.7 ± 5.6	66.5 ± 6.1	67.4 ± 5.8	0.936
E/A	1.16 ± 0.36	1.25 ± 0.30	1.12 ± 0.40	0.95 ± 0.25	0.035
Deceleration time, ms	223 ± 47	225 ± 52	215 ± 40	260 ± 40	0.037
Left atrial size, mm	35.4 ± 4.5	34.5 ± 4.4	36.0 ± 4.8	37.2 ± 2.2	0.116
E/E’	8.77 ± 2.26	6.83 ± 0.81	9.69 ± 1.26	14.01 ± 1.13	< 0.001
Mean baPWV, cm/s	1,325 ± 185	1,261 ± 163	1,345 ± 169	1,569 ± 232	< 0.001

LDL, low density lipoprotein; HDL, high density lipoprotein; baPWV, brachial-ankle pulse wave velocity.

*results from the comparisons among subjects with E/E’ < 8, 8–12.9 and ≥ 13.

**Figure 1:**
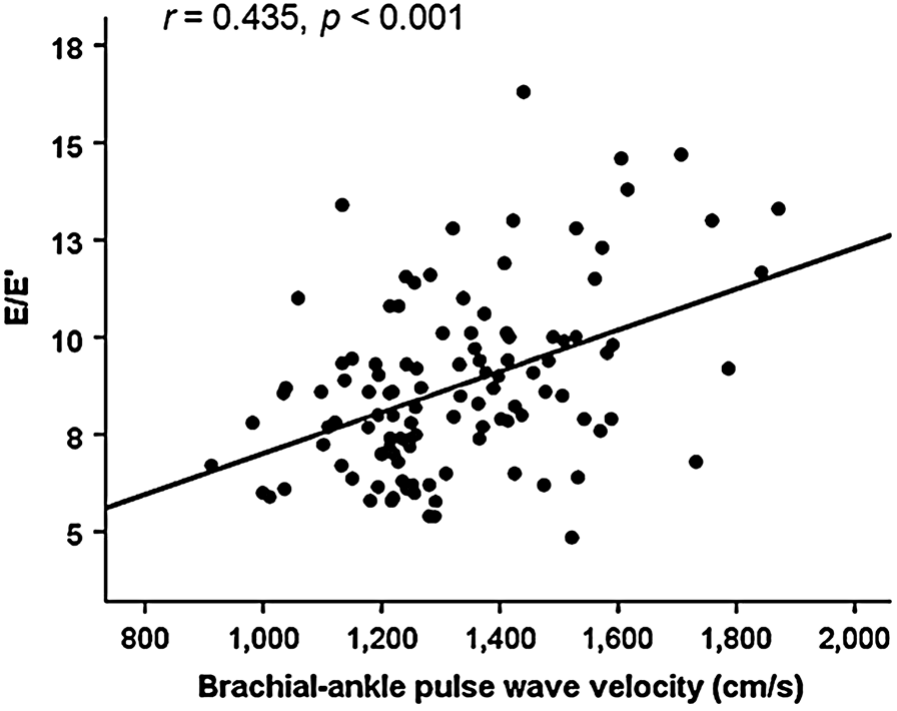
**Scatter plot showing the association between baPWV and E/E’.** BaPVW, brachial-ankle pulse wave velocity.

**Figure 2:**
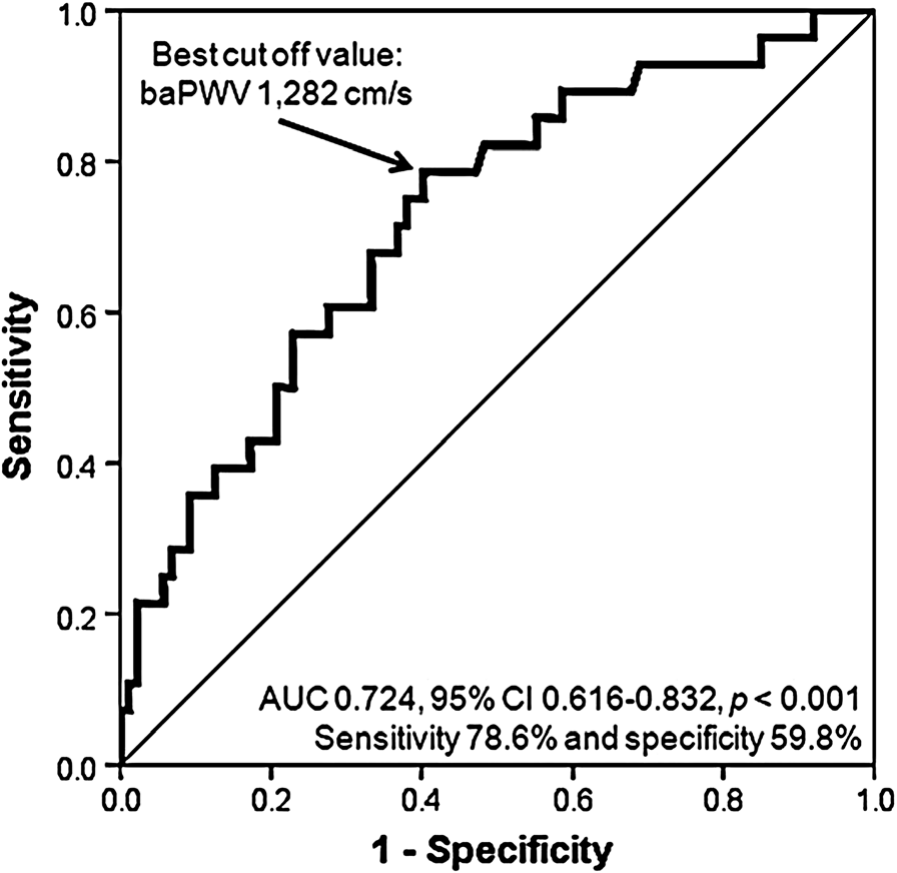
**ROC curve of baPWV for prediction of E/E’ ≥ 10.** ROC, receiver operating characteristics; baPVW, brachial-ankle pulse wave velocity. AUC, area under curve; CI, confidence interval.

**Figure 3:**
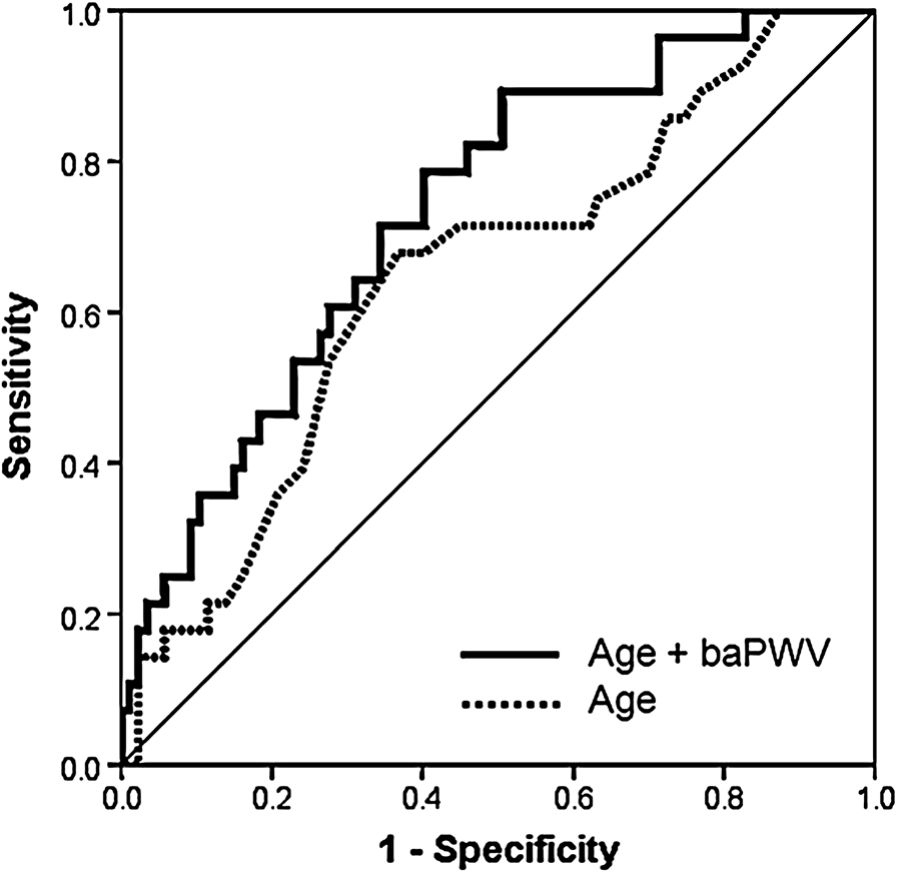
**Incremental ROC analysis for prediction of E/E’ ≥ 10.** ROC, receiver operating characteristics; baPVW, brachial-ankle pulse wave velocity.

In order to identify independent predictors of E/E’, multiple linear regression analysis was performed (Table 2). Gender stratified analysis was also performed in this multivariable model. Sex (*β* = 0.238*, p* = 0.004*)*, body mass index (*β* = 0.220*, p* = 0.007*)* and baPWV (*β* = 0.371*, p* < 0.001*)* were independently associated with E/E’ in a total subjects. In gender stratified analysis, baPWV was independently associated with E/E’ in men (*β* = 0.417*, p* < 0.001*)* but not in women (*β* = 0.204*, p* = 0.228*)*. Age had a significant association with E/E’ only in women (*β* = 0.379*, p* = 0.026*).* The correlation of baPWV with E/E’ was shown using a scatter plot in Figure 1 (*r* = 0.435, *p* < 0.001)**.** In ROC curve analysis, the sensitivity and specificity for detection of E/E’ ≥ 10 were 78.6% and 59.8%, respectively with mean baPWV of 1,282 cm/s as the cut off value (Figure 2). The discriminatory capacity for predicting E/E’ ≥ 10 was improved from an area under the ROC curve of 0.646 with age alone to 0.734 when baPWV was added (*p* < 0.001) (Figure 3).

**Table 2:** Independent associations between v and E/E’

**Variable**	**Total**		**Men**		**Women**	
	***β***	***p***	***β***	***p***	***β***	***p***
**Age**	0.090	0.303	- 0.024	0.828	0.379	0.026
**Sex**	0.238	0.004	-	-	-	-
**Body mass index**	0.220	0.007	0.160	0.131	0.238	0.112
**BaPWV**	0.371	< 0.001	0.417	< 0.001	0.204	0.228

BaPWV, brachial-ankle pulse wave velocity.

Some other parameters representing LV diastolic functions including A wave velocity, E/A, E’ velocity and E’/A’ also had significant associations with baPWV in univariate analyses, however its significance was vanished after controlling of potential confounders including age, sex and body mass index (data not shown).

## Discussion

In this study, we showed that baPWV is independently associated with E/E’ among apparently healthy Korean individuals, even after controlling of age, sex and body mass index. To the best of our knowledge, this is the first report showing the association between baPWV and E/E’ in healthy subjects. Our result suggests close interactions between aortic stiffness and LV filling pressure and usefulness of baPWV for early detection and prevention of increased LV filling pressure.

Previously, most studies have investigated the correlation between LV diastolic function and arterial stiffness in some patient groups with hypertension, diabetes, renal failure or coronary artery disease [14-20,24]. Wang et al. reported that arterial stiffness measured by baPWV is independently and negatively associated with E’ velocity in hypertensive subjects [19]. Eren et al. demonstrated significant correlations between aortic distensibility and deceleration time of E wave among patients with hypertension, diabetes, or both [14]. In one Korean report, baPWV is independently correlated with E/A ratios in women undergoing hemodialysis [16]. A few studies have been performed to investigate the association between diastolic function and arterial stiffness among general populations without limitation to special population of certain medical conditions. Redfield et al. reported age-dependent increase in both E/E’ and vascular stiffening in a community-based adults without cardiovascular disease [25]. However, subjects with hypertension, diabetes and structural heart disease were enrolled, vascular stiffness was indirectly estimated using blood pressure and stroke volume, and the direct relationship between E/E’ and vascular stiffening was not shown in that study. Abhayaratna and colleagues demonstrated that age-related deterioration in diastolic dysfunction is independently associated with increased aortic stiffness among participants of population-based echocardiographic survey [26]. They showed independent relationship between carotid-femoral pulse wave velocity (cfPWV) and diastolic function grade, however direct relationship between cfPVW and E/E’ was not demonstrated in that study. Also, they included the subjects with hypertension, diabetes and coronary artery disease in the analyses. Xu et al. showed that baPWV was significantly associated with E/A ratio among healthy adults with a normal LV ejection fraction [27]. Other report demonstrated that central carotid stiffness is associated with E’ velocity among healthy subjects [28]. All these previous findings are in line with our results showing the association between arterial stiffness and LV diastolic function. As compared to previous studies, we think that our study results may have a possible novelty because we focused on the direct relationship between baPWV and E/E’ and confirmed its independent associations in an apparently healthy population without known cardiovascular risk factors or overt heart disease.

Measuring LV filling pressure is a critical component for the diagnosis of diastolic dysfunction [29]. Although cardiac catheterization is the gold standard in measuring LV filling pressure, its risk of invasive measurement is a major concern. Recent studies have shown similar efficacy and outcomes between the non-invasive measurement of LV filling pressure by echocardiography and invasive measurement by Swan-Ganz catheter [30]. E/E’ measured by TDI technique of transthoracic echocardiography provides better estimates of LV filling pressure than other echocardiographic methods such as pulmonary vein and preload reduction, therefore it has been considered as the best index for LV filling pressure and diastolic function among non-invasive measurements [30,31].

Several studies have reported the relationship between arterial stiffness and E/E’. Shim et al. showed that pulse pressure amplification is significantly associated with E/E’ in women without known cardiovascular risk factors but not in men [32]. Vriz et al. investigated the association between carotid artery stiffness and diastolic function in 92 healthy subject, but E/E’ was not associated with carotid artery stiffness in that study [28]. A study in Australia showed a significant association between arterial stiffness measured by central pulse pressure and E/E’ among 70 patients with hypertension, however the significant correlation was shown only in univariate analysis [15]. In our study, there was a significant correlation between baPWV and E/E’ in a healthy population, even when we controlled potential confounders.

It should be addressed here about the sex difference in the association between baPWV and E/E’. In the present study, we demonstrated that baPWV is independently associated with E/E’ in men but not in women. This result suggests the sexual differences in central hemodynamics. A few previous studies have investigated the sex difference in the relationship between arterial stiffness and LV diastolic dysfunction [26,32]. Shim et al. reported that E/E’ was significantly associated with pulse pressure amplification in women but not men, but there were no correlations between E/E’ and cfPWV in both sexes [32]. On the contrary, Abhayaratna et al. showed significant association between aortic PWV and E/E’ in both sexes in univariate analyses [26]. We think that different study populations and tools measuring arterial stiffness may contribute to the different results. Further studies will be needed to demonstrate a firm conclusion.

There are several plausible pathways that might explain the association between increased arterial stiffness and LV filling pressure. Increased arterial stiffness causes premature return of the reflected pulse wave in the last systole. Its arrival during LV ejection may lead to augmentation of the central aortic pressure wave amplitude, thus increasing aortic systolic blood pressure and decreasing aortic diastolic pressure. The resultant increase in afterload during LV systole and reduction coronary perfusion during LV diastole may lead to promote LV concentric remodeling and hypertrophy and may also directly slow LV relaxation [15,33]. Indeed, increased LV filling pressure in association with arterial stiffening has been shown in patients with overt diastolic dysfunction, and interaction of these processes may be important in the clinical expression of heart failure [34].

LV filling pressure provides valuable information for prediction of clinical outcomes of patients with various heart diseases. Hills et al. reported that E/E’ is a strong predictor of mortality in patients after acute myocardial infarction [35]. McMahon and colleagues showed that E/E’ predicts adverse clinical outcomes in children with hypertrophic cardiomyopathy [36]. More recently, Hirata et al. reported that a combined information of LV systolic function and E/E’ allowed the identification of patients at higher risk of readmission and cardiac death in patients with heart failure [37]. In the general population, one study reported that E’ is a powerful and independent predictor of death, however E/E’ is not associated with mortality [25]. E/E’ is also important in terms of diagnosis of heart failure with preserved ejection fraction (HFpEF). About 50% of heart failure is HFpEF, and the importance of HEpEF is becoming recognized increasingly. Recent guidelines have focused on the evaluation of LV filling pressure for the diagnosis of HFpEF, and evaluation of LV filling pressure using Doppler echocardiography is currently regarded as an essential component for grading diastolic function in patients with suspected HFpEF [29,38]. In the present study, we showed that the baPWV cut off value of 1,282 cm/s is useful in estimation of increased LV filling pressure (E/E’ ≥ 10) by ROC curve analysis. In addition, baPWV had an incremental value over age for the detection of E/E’ ≥ 10. These findings suggest that simple and non-invasive measurement of baPWV enables us to detect increased LV filling pressure and diastolic dysfunction before they develop clinically relevant heart failure especially among healthy population.

Robust evidence suggests that increased arterial stiffness is associated with adverse cardiovascular outcomes in general population [39,40]. Recent meta-analysis including more than 15,000 subjects confirmed that PWV is an independent predictor of cardiovascular events and all-cause mortality. They showed that an increase of arterial PWV of 1 m/s raise cardiovascular risk by more than 10% [13]. Accordingly, high baPWV is not only a prognostic factor for clinical outcomes by itself but also represents high LV filling pressure, both of which have significant prognostic impacts on clinical outcomes.

Some previous studies have used cfPWV for measurement of arterial stiffness, which represents more selective central arterial wall stiffness rather than muscular arterial stiffness [41]. On the other hand, recently developed baPWV measurements reflects arterial stiffness in the central and peripheral arteries between the brachial and ankle arteries [6]. Therefore, there had been a concern whether baPVW could play a role instead of cfPWV. However, baPWV is easier to perform than cfPWV, therefore it is more commonly used in clinical practice, and its usefulness and prognostic value have been proven in various clinical settings [7-12] and meta-analysis [3,13]. In addition, it has been reported that baPWV shows good correlation with cfPWV [42] and aortic stiffness obtained by invasive recording [6]. Accordingly, baPWV can be considered an acceptable marker of arterial stiffness with an efficacy comparable to that of cfPWV.

Several limitations of this study should be considered. First, this study was conducted with cross-sectional design, and there is a possibility of a temporal relationship between arterial stiffness and E/E’. Second, study sample size was small. Lastly, TDI was measured only at the medial side. A better relation might have been found between lateral TDI and baPWV.

## Conclusions

The present study shows a positive association between baPWV and E/E’ among healthy Korean population without known cardiovascular risk factors or overt cardiovascular disease. This result suggests close interactions between aortic stiffness and LV filling pressure. BaPWV is useful as a simple and non-invasive method for early detection of increased LV filling pressure and diastolic dysfunction among healthy individuals. Further prospective investigations with a large sample size are needed to confirm our results.

## Abbreviations

AUC: Area under curve; BaPWV: Brachial-ankle pulse wave velocity; cfPWV: Carotid-femoral pulse wave velocity; CI: Confidence interval; DT: Deceleration time; HFpEF: Heart failure with preserved ejection fraction; LV: Left ventricle; OR: Odds ratio; ROC: Receiver-operating characteristic; SD: Standard deviation; TDI: Tissue Doppler imaging.

## Competing interest

The authors declare that they have no competing interests.

## Authors’ contributions

The work presented here was carried out in collaboration between all authors. H-LK and M-SI analyzed data and wrote the paper; J-HZ designed the study, interpreted the results, wrote the paper and had all responsibilities of this work; J-BS, W-YC, S-HK, and M-AK were involved in study design and interpretation of data. All authors read and approved the final manuscript.
